# Human Survivin and *Trypanosoma cruzi* Calreticulin Act in Synergy against a Murine Melanoma *In Vivo*

**DOI:** 10.1371/journal.pone.0095457

**Published:** 2014-04-22

**Authors:** Lorena Aguilar-Guzmán, Lorena Lobos-González, Carlos Rosas, Gerardo Vallejos, Cristián Falcón, Eduardo Sosoniuk, Francisca Coddou, Lisette Leyton, David Lemus, Andrew F. G. Quest, Arturo Ferreira

**Affiliations:** 1 Program of Immunology, Institute of Biomedical Sciences, Faculty of Medicine, University of Chile, Santiago, Chile; 2 Laboratory of Cellular Communication, Center for Molecular Studies of the Cell, Program of Cell and Molecular Biology, Institute of Biomedical Sciences, Faculty of Medicine, University of Chile, Santiago, Chile; 3 Program of Anatomy and Developmental Biology, Institute of Biomedical Sciences, Faculty of Medicine, University of Chile, Santiago, Chile; Baylor College of Medicine, United States of America

## Abstract

Immune-based anti-tumor or anti-angiogenic therapies hold considerable promise for the treatment of cancer. The first approach seeks to activate tumor antigen-specific T lymphocytes while, the second, delays tumor growth by interfering with blood supply. Tumor Associated Antigens are often employed to target tumors with therapeutic drugs, but some are also essential for tumor viability. Survivin (Surv) is a member of the inhibitor of apoptosis protein family that is considered a Tumor Associated Antigen important for cancer cell viability and proliferation. On the other hand, *Trypanosoma cruzi* (the agent of Chagas’ disease) calreticulin (TcCRT) displays remarkable anti-angiogenic properties. Because these molecules are associated with different tumor targets, we reasoned that immunization with a Surv-encoding plasmid (*pSurv*) and concomitant TcCRT administration should generate a stronger anti-tumor response than application of either treatment separately. To evaluate this possibility, C57BL/6 mice were immunized with *pSurv* and challenged with an isogenic melanoma cell line that had been pre-incubated with recombinant TcCRT (rTcCRT). Following tumor cell inoculation, mice were injected with additional doses of rTcCRT. For the combined regimen we observed in mice that: **i).** Tumor growth was impaired, **ii).** Humoral anti-rTcCRT immunity was induced and, **iii).** *In vitro* rTcCRT bound to melanocytes, thereby promoting the incorporation of human C1q and subsequent macrophage phagocytosis of tumor cells. These observations are interpreted to reflect the consequence of the following sequence of events: rTcCRT anti-angiogenic activity leads to stress in tumor cells. Murine CRT is then translocated to the external membrane where, together with rTcCRT, complement C1 is captured, thus promoting tumor phagocytosis. Presentation of the Tumor Associated Antigen Surv induces the adaptive anti-tumor immunity and, independently, mediates anti-endothelial cell immunity leading to an important delay in tumor growth.

## Introduction

About 7.6 million people die every year from cancer, accounting for 13% of the total disease-caused mortality world-wide. Notably, 70% of these deaths occur in middle- or low-income countries [1]. In cancerous cells a variety of genomic changes occur to facilitate self-sufficiency in growth signals, reduce sensitivity to anti-growth signals, and mediate unlimited growth, abnormal tissue invasiveness and metastasis. These changes also help evade apoptosis and produce pro-angiogenic molecules, two important cancer-related processes where Survivin (Surv) and Calreticulin (CRT) participate among many other proteins [2].

Surv, the smallest member of the inhibitors of apoptosis family (IAP), contains a single BIR domain [3] and it is overexpressed in most human cancers, where it reduces apoptosis and promotes cell proliferation [4]. The Surv gene (*Surv*), represents the fifth most highly expressed tumor-associated antigen (TAA) [5] and encodes a 142 aa, 16.5 kDa protein [6] that is expressed in development but not in normal adult tissues, except transiently in proliferating cell populations [7], [8]. Surv is thus considered a potential molecular target in cancer treatment [7], [9]. Surv also plays an important role in endotheliocytes, although expression is lower than in transformed cells [7], [8]. Thus, Surv may be considered an ideal target for a DNA vaccine that triggers a T cell response, which will affect both the tumor cells and the tumor vasculature [10]. When human Surv cDNA (from a colon adenocarcinoma, HT-29), cloned into a *pcDNA3.1* vector (*pcDNA3.1-Surv or pSurv*) was injected into Balb/c mice (together with a *pGM-CSF* plasmid), expression of Surv protein and derived MHC-I associated peptides was observed. This was followed by an interesting anti-Surv cellular immune response [11].

CRT, on the other hand, is a pleiotropic and evolutionarily conserved, endoplasmic reticulum (ER)-resident chaperone, present in all nucleated cells, on the cell surface and in the extracellular milieu [12]–[15]. CRT participates in the regulation of calcium homeostasis and as a chaperone in the folding of new glycoproteins. The importance of CRT is evident in “knock out” mice that die *in utero* 14.5 to 16.5 days after fertilization, due to incorrect cardiac development and to deregulation of calcium homeostasis in the endoplasmic reticulum [16]. CRT from vertebrates interferes with the binding of endothelial cells to extracellular matrix components [17], modulates gene expression, phagocytosis of apoptotic cells, and inhibits the C1q-dependent activation of the complement system [18]. Moreover, CRT and its 180 amino acid fragment from the amino terminus (vasostatin) inhibit cell proliferation, angiogenesis and tumor growth [19], [20]. Anti-tumor drugs such as anthracyclines and oxaliplatin, or ionizing radiation, result in CRT translocation to the tumor cell surface, immunogenic cell death and phagocytosis by dendritic cells [21]–[23]. In general, enhanced CRT translocation to the surface, with resulting increased immunogenicity is observed in tumor cells exposed to stress situations. In general, enhanced CRT translocation to the surface results in increased immunogenicity of tumor cells exposed to stress situations [24]–[27].

We have described that *Trypanosoma cruzi* Calreticulin (TcCRT), similar to its counterpart from vertebrates, translocates from the ER to the parasite surface. There, TcCRT inhibits the classical and lectin pathways of human complement activation [18], [28], promotes parasite infection [29], [30], as well as inhibits angiogenesis [31], [32] and tumor growth in several *in vivo*, *ex vivo* and *in vitro* experimental set ups [31]–[33]. The anti-angiogenic and anti-tumor effects of TcCRT are, in general, more potent than those elicited with human CRT (HuCRT) at equimolar concentrations. Both, the intra and extracellular TcCRT functions seem to be vital for the parasite, since only those cells with hemiallelic TcCRT gene inactivation survive [34].

The inhibition of the angiogenic process has been used as an approach in cancer therapy, since most solid tumors are highly vascular and thus vulnerable to decreased blood supply. For this reason, approaches aimed at suppressing angiogenesis are applicable to a wide variety of tumors. Given the low mutagenic potential of endothelial cells, such approaches are less likely to result in resistance to treatment [35].

The ability of TcCRT to inhibit angiogenesis, may generate a state of stress in tumor cells. As a consequence, translocated endogenous CRT to the cell surface acts as a C1 receptor [24]–[27]. The high sequence homology of CRT among different species, particularly in the vasostatin-like fragment, explains the conserved function of the protein in extremely evolutionarily distant organisms, including protozoan pathogens. Thus, there is 46% identity and 60% homology between the anti-angiogenic fragment of HuCRT (aa 120–180) [18] and that of the *T. cruzi* counterpart (aa 136–191) [31]. The available experimental evidence identifies as a putative TcCRT molecular target, a scavenger receptor - type I expressed by endothelial cell, since the interaction is inhibited by fucoidin, an algal sulfated fucose-based polysaccharide [31]. On the other hand, genetic immunization with *Surv* should target surface Surv-expressing endotheliocytes. A synergistic effect of both procedures is to be expected.

In synthesis, here we provide evidence that immunization with human *Surv*, together with systemic rTcCRT administration, synergistically inhibit tumor growth in a murine melanoma model by acting on different molecular targets.

## Materials and Methods

### Animal Model

C57BL/6 female 6–8 week old mice were bred and maintained at the Animal Facility (Disciplinary Program of Immunology, Institute of Biomedical Sciences, Faculty of Medicine, University of Chile). Experiments were performed in compliance with the ‘Guide for the Care and Use of Laboratory Animals’, National Research Council, Washington DC, USA, 2002 [36]. All procedures were approved by the local Bioethics Committee (Bioethics Committee, Faculty of Medicine, University of Chile) (Approval Identification Number: CBA# 0278 FMUCH). The *pSurv* and rTcCRT effects on the *in vivo* growth of a murine melanoma were assessed in two independent sets of experiments, using five mice in each one.

### Cells

B16-F10, derived from a spontaneously occurring murine melanoma, were cultured in RPMI media, supplemented with fetal bovine serum (FBS) 10% (v/v), 100 IU/ml Penicillin and 100 µg/ml Streptomycin (Gibco BRL, Life Technologies, Grand Island, NY, USA) at 37°C, with 5% CO_2_.

### Proteins

rTcCRT, its R domain (aa 136–281), and rHuCRT were cloned and purified as described [18].

### *In* *vivo* Assays

On days 1 and 7 the animals were anesthetized with a combination of Ketamine (100 mg/Kg) and Xylazine (10 mg/kg), i.p. and then injected into the footpads, with 100 µg of plasmidial DNA (Qiagen Maxi Prep), using TLRs Ligands CpG ODN (5′T*C*C*A*T*G*A*C*G*T*T*C*C*T*G*A*T*G*C*T*3′) (Integrated DNA Technologies, USA; *Phosphorothioate bonds) and Poly I:C (Amersham Bioscience, USA), 20 and 10 µg/animal, respectively, in 100 µl total volume.

On day 14, mice were challenged s.c. with 100 µl containing 3×10^5^ B16-F10 syngeneic melanoma cells, pre-incubated with either 100 µg rTcCRT, its R domain or rHuCRT, for 30 min at 37°C. (These treatments will be referred below as *pSurv-*rTcCRT or *pSurv*-rHuCRT, when recombinant HuCRT is used instead of the parasite molecule).

On days 16–30, tumor growth was evaluated and 100 µg of the proteins were administrated s.c. (peri tumor infiltration) every other day. According to established bioethical regulations, tumors were measured until they reached a maximum of 3,000 mm^3^ (π/6×length×width^2^) [37], when the animals were euthanized.

### Immunohistochemistry

Tumors were fixed in 0.1 M phosphate buffer (pH 7.3)/formaldehyde 10% (v/v) for 48 h, dehydrated in alcohol, clarified in Xylene, embedded in paraffin, and sectioned at 5 µm (Microtome Leitz 1512). Paraffin histological sections were stained with hematoxylin-eosin for routine histological analysis. In order to detect blood vessels, standard immunohistochemistry was used. Briefly, histological sections were treated with methanol/hydrogen peroxide 3% (v/v) for 10 min and incubated for 30 min in Dako Target Retrieval (Dako, Carpinteria, CA, USA) on a steamer, and blocked using a HistoMouse-MAX kit (Invitrogen, CA, USA). The tissue was probed with a rat anti-mouse CD31-PECAM antibody (Hycult biotec), followed by a goat anti-rabbit IgG conjugated to peroxidase to which a commercially available substrate was added (Histomouse MAX-AEC Broad Spectrum Kit) (Invitrogen, Camarillo, CA, USA).

### Binding of rTcCRT to B16-F10 Cells

3×10^5^ melanoma cells were incubated with 2.5–100 µg rTcCRT, for 30 min at 37°C. A rabbit polyclonal antiserum against the parasite protein or, as a negative control, a pre- immune serum (from a rabbit bled prior to the rTcCRT immunization) were used to determine the rTcCRT presence on the cell membranes, followed by an anti-rabbit FITC-conjugated goat antibody, detected by flow cytometry.

C1q binding to rTcCRT (4 µg/3×10^5^ cells) was detected with FITC-conjugated rabbit antibodies against C1q, after incubation for 30 min at 37°C. C1q binding was blocked by incubating with F(ab’)_2_ antibody fragments against rTcCRT, in 1.5, 3.0 and 6.0 µg/3×10^5^ cells, followed by C1q incubation and antibody detection.

Finally, as a control, cells were incubated with a rabbit antibody against *Mus musculus* CRT (MmCRT) and a FITC-conjugated goat antibody against rabbit IgG (evaluated by flow cytometry).

### Phagocytosis Assay

B16-F10 melanoma cells were incubated for 10 min at 37°C with 5,6 carboxyfluorescein diacetate succinimidyl ester (CFS-E) (Sigma-Aldrich) at 2.5 mM, in RPMI without serum. The cells were then washed twice with RPMI-FBS 20% (v/v), incubated with 40 µg of rTcCRT and finally with 4 µg of C1q for 30 min at 37°C. On the other hand, the murine macrophage cell line, RAW 264.7, was probed using a rabbit anti-mouse CD14, conjugated with Phycoerythrin (PE), at a 1/10 dilution, for 1 h, at 37°C. Both labeled cells were incubated at 37°C for 3 h, and analyzed by flow cytometry.

### ELISA

Plates coated overnight at 4°C with 500 ng rTcCRT/well in carbonate buffer, were blocked with 200 µg BSA/well in PBS, washed with 0.05% (v/v) Tween 20 in PBS (PBS-Tween 20) and incubated for 90 min at 37°C (Dynamic Incubator, Abbott). The plates were then washed and incubated with antisera, diluted 1/100, from rTcCRT or *pSurv*-rTcCRT immunized mice, for 90 min at 37°C. After washing, the plates were incubated with a sheep anti-mouse IgG peroxidase conjugate (DAKO) (1/1.000), for 90 min at 37°C. After washing, the reaction was developed with a substrate solution for peroxidase (ABTS) and samples were evaluated at 405 nm (Microplate Reader, Bio-Rad). As negative and positive controls, pre-immune sera from each mouse (serum from mice bled prior to genetic immunization and rTcCRT administration), and the E2G7 monoclonal anti-rTcCRT antibody, were respectively used.

### Statistical Analysis

Using the GraphPad Prism 5 program, tumor growth curves were validated with the Wilcoxon Signed Rank test. Animal survival in each group was evaluated using the Mantel Cox test and phagocytic indexes were validated with a t test. The Mann Whitney test was used to evaluate individual tumor growth, the number of blood vessels in tumor histological sections and the immune humoral responses. P values≤0.05 were considered as statistically significant.

## Results

### Combined of *pSurv* Genetic Immunization, in Conjunction with rTcCRT, Delays the Growth of B16-F10 Murine Melanoma

Initially, we determined whether rTcCRT, *per se*, affects the *in vitro* tumor cell proliferation. We observed that B16-F10 cell proliferation was similar in rTcCRT presence or absence (data not shown).

**Figure 1:**
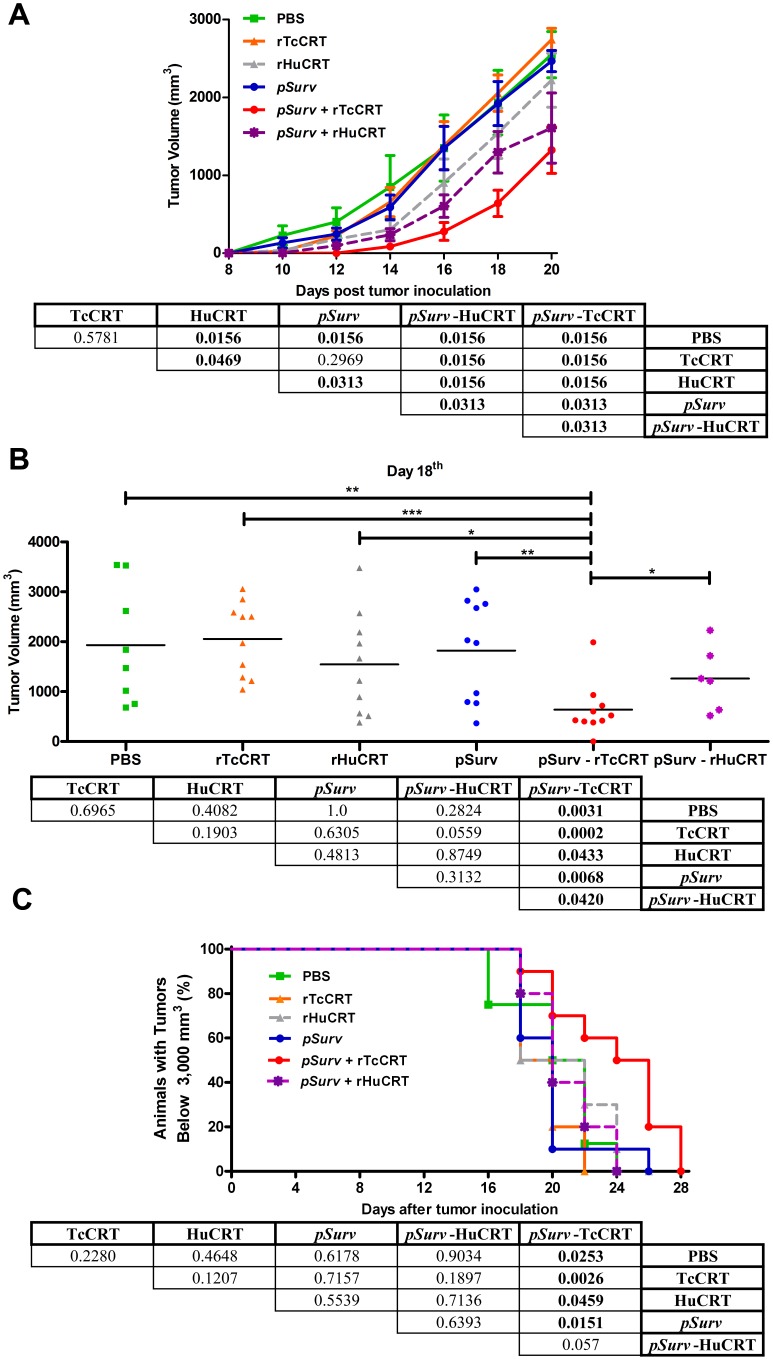
Genetic immunization with *pSurv*, along with rTcCRT, slows the growth of an experimental murine melanoma. Mice were immunized with *pSurv* plasmid and then inoculated s.c. with PBS, rTcCRT or rHuCRT or each one of them alone. A: Time-course of the effects on tumor growth of the different treatments. B: Distribution measurements of solid tumors in each group, evaluated at day 18 (See A) after injection of tumor cells. C: Survival curve recorded as a percentage of surviving animals (those bearing 3,000 mm^3^ tumors were euthanized, according to bioethical considerations) until day 28 post-challenge with tumor cells. In A–C, the bottom panel shows p values (significant ones in bold) for all relevant comparisons.

Since rTcCRT did not affect tumor cell proliferation, we performed the assay using genetic immunization with *Surv* (*pSurv*) together with rTcCRT s.c. inoculation (peri tumor infiltration) (As mentioned in the Methods section, these treatments are referred below as *pSurv-*rTcCRT or *pSurv*-rHuCRT, when recombinant HuCRT is used instead of the parasite molecule) (Figure 1). rHuCRT was included because anti-angiogenic effects are reduced compared to rTcCRT [31]. The top panel in Figure 1A shows the tumor growth curves of the 6 groups of animals, subjected to different treatments. Three distinct groups in terms of tumor growth were identified: **i).** A higher tumor growth was observed in the animals treated with PBS, rTcCRT and *pSurv*. While no differences were detected when the rTcCRT/PBS or rTcCRT/*pSurv* groups were compared, the *pSurv* group showed a reduced tumor growth rate, when compared with PBS-treated animals; **ii).** Animals treated only with *pSurv*-rHuCRT or rHuCRT alone, showed an intermediate tumor growth rate. The growth rate is different between these groups, but lower than those observed in i). **iii).** Finally, and most importantly, the group treated with the *pSurv-*rTcCRT combination shows synergistic behavior where the highest delays in tumor growth were detected. These observations were statistically validated as shown in the bottom panel of Figure 1A.

We then compared the tumor volumes of each animal treated with the different protocols at day 18 (Figure 1B, top panel). Again, the group treated with *pSurv* immunization and rTcCRT parenteral administration developed the smallest tumors. The animals behaved more homogeneously than those treated otherwise. Statistical validation of these two conclusions is presented in Figure 1B (bottom panel).

Animal survival was quantified following ethical considerations establishing that a maximum of 3,000 mm^3^ tumor growth is allowed, at which point the animals must be euthanized. Results of animal survival, measured under these conditions, and statistical validation are summarized in Figure 1C.

### *pSurv* Immunization, in Conjunction with the rTcCRT-R Domain Administration, Slows Murine Melanoma Growth

**Figure 2:**
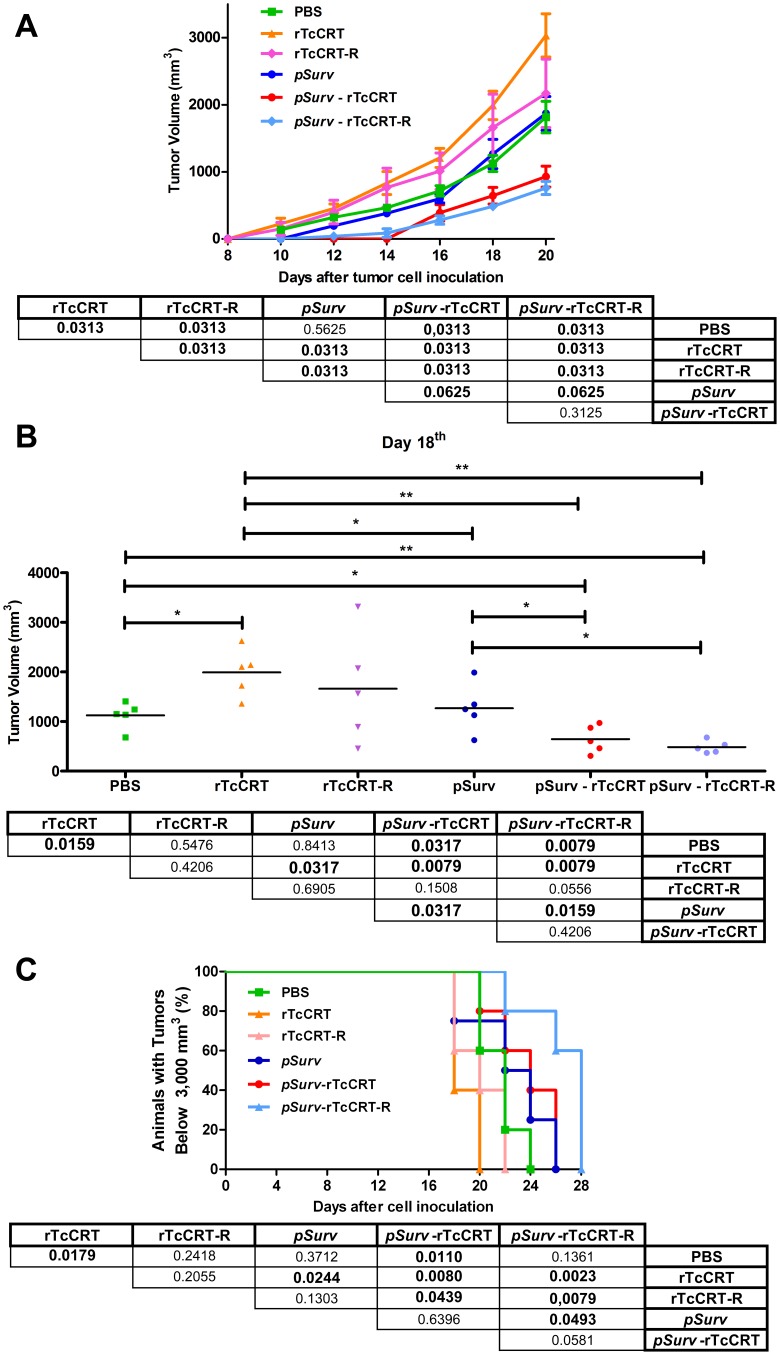
Genetic immunization with *pSurv*, together with the rTcCRT-R, retards the growth of experimental murine melanoma. A: Tumor growth curve. B: Distribution measurements of solid tumors in ten animals in each group, at day 18 (See A) after injection of tumor cells. C: Survival curve recorded as a percentage of surviving animals until day 28 post-challenge with tumor cells. In A–C, the bottom panel shows p values (significant ones in bold) for all relevant comparisons.

The rTcCRT-R domain (aa 136–281), previously characterized in proliferation assays and capillary morphogenesis *in vitro* with HUVEC cells, does not inhibit or affect angiogenesis [31]. As observed for the whole parasite molecule (Figure 1), rTcCRT-R alone did not inhibit tumor growth. However, the combination of *pSurv* genetic immunization with conventional rTcCRT-R administration (Figure 2A), slows tumor formation to a similar extent as observed before with the *pSurv*-rTcCRT combination (Figure 1A, top panel). These conclusions are statistically confirmed in Figure 2A, bottom panel.

Likewise, on day 18 post B16-F10 inoculation, in animals treated with the *pSurv*-rTcCRT-R combination, tumor growth was more effectively inhibited, as compared to other treatments (Figure 2B). Furthermore, *pSurv*-rTcCRT-R induced a lower tumor growth than that observed in animals treated with PBS, rTcCRT, rTcCRT-R and *pSurv*, as confirmed by statistical analysis (Figure 2B, bottom panel).

Animal survival, quantified as described above, and statistical validations are summarized in Figure 2C.

### Experimental Treatment with rTcCRT and *pSurv*, Combined or Alone, Inhibit Tumor Angiogenesis

**Figure 3:**
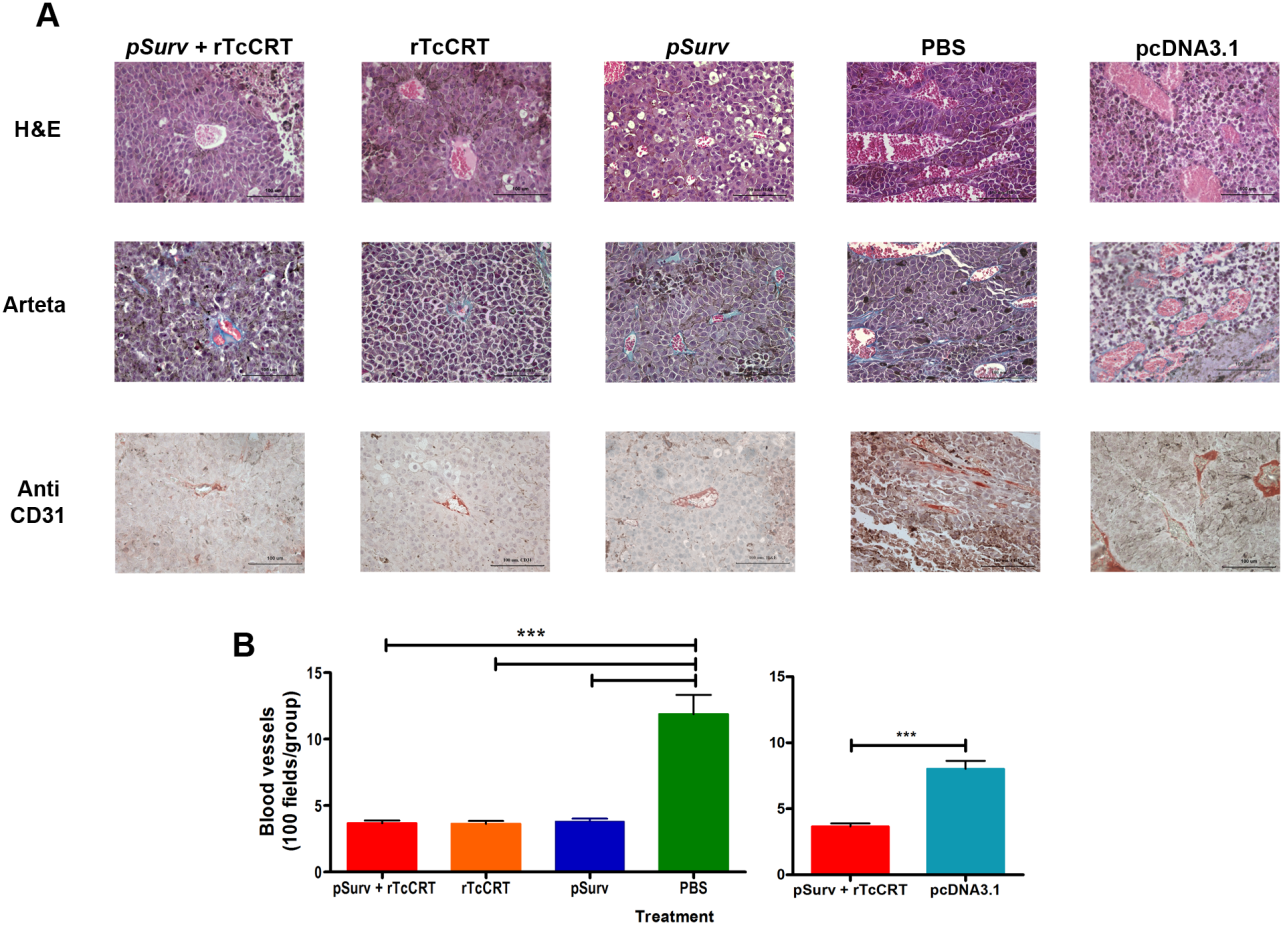
Immunization with *pSurv* and inoculation with rTcCRT, together or separately, are anti-angiogenic. A: Hematoxylin-eosin (HE) (first row) or Arteta (second row) staining and immunohistochemistry using anti-CD31 antibody (third row). B: Double-blind angiogenesis quantification in tumors sections of a murine melanoma.

Double blind quantification of tumor vascularization (stroma and parenchyma) was assessed by labeling the vessels with anti-CD31 antibody and contrasting using Arteta staining, specific for collagen (Figure 3A). Treatment with *pSurv* and/or with rTcCRT inoculation did inhibit tumor angiogenesis. On the other hand, animals treated with the empty plasmid or vehicle, developed tumors with normal frequency and large caliber and tortuous capillaries developed (Figure 3B). No differences were observed in the irrigation of tumors obtained from animals treated with *pSurv* or rTcCRT or both. In these three cases a lower number of vessels were detected, as compared with the control groups treated with PBS or empty plasmid.

### rTcCRT Binds to B16-F10 Melanocytes

**Figure 4:**
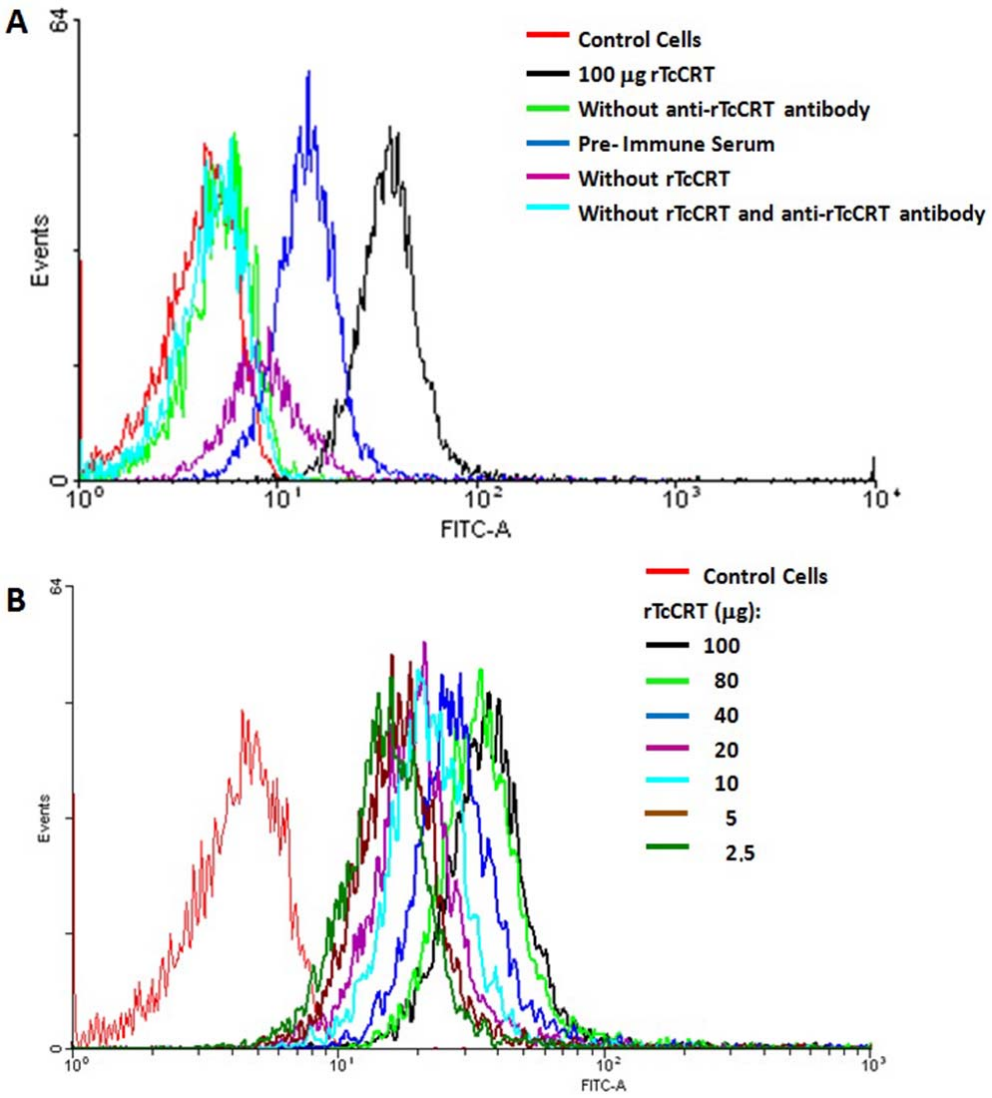
rTcCRT binds to B16-F10 cells. rTcCRT binding to B16-F10 melanocytes was evaluated by flow cytometry against polyclonal antibodies to the recombinant protein. A: Single concentration rTcCRT binding to melanocytes B: rTcCRT dose-response binding to melanocytes.

To determine the effect of TcCRT on tumor cells, we first evaluated rTcCRT binding capacity to melanoma cells. rTcCRT is not toxic *per se* to B16-F10 melanocytes (not shown), although it binds, in a dose-dependent manner, to the tumor cell surface, as shown by flow cytometry (Figure 4). Indeed, surface labeling of B16-F10 cells with rTcCRT was observed at concentrations similar to those used *in vivo* (Figure 4).

### C1q, the First Component of Human Complement Classical Pathway, Binds to Melanocytes

**Figure 5:**
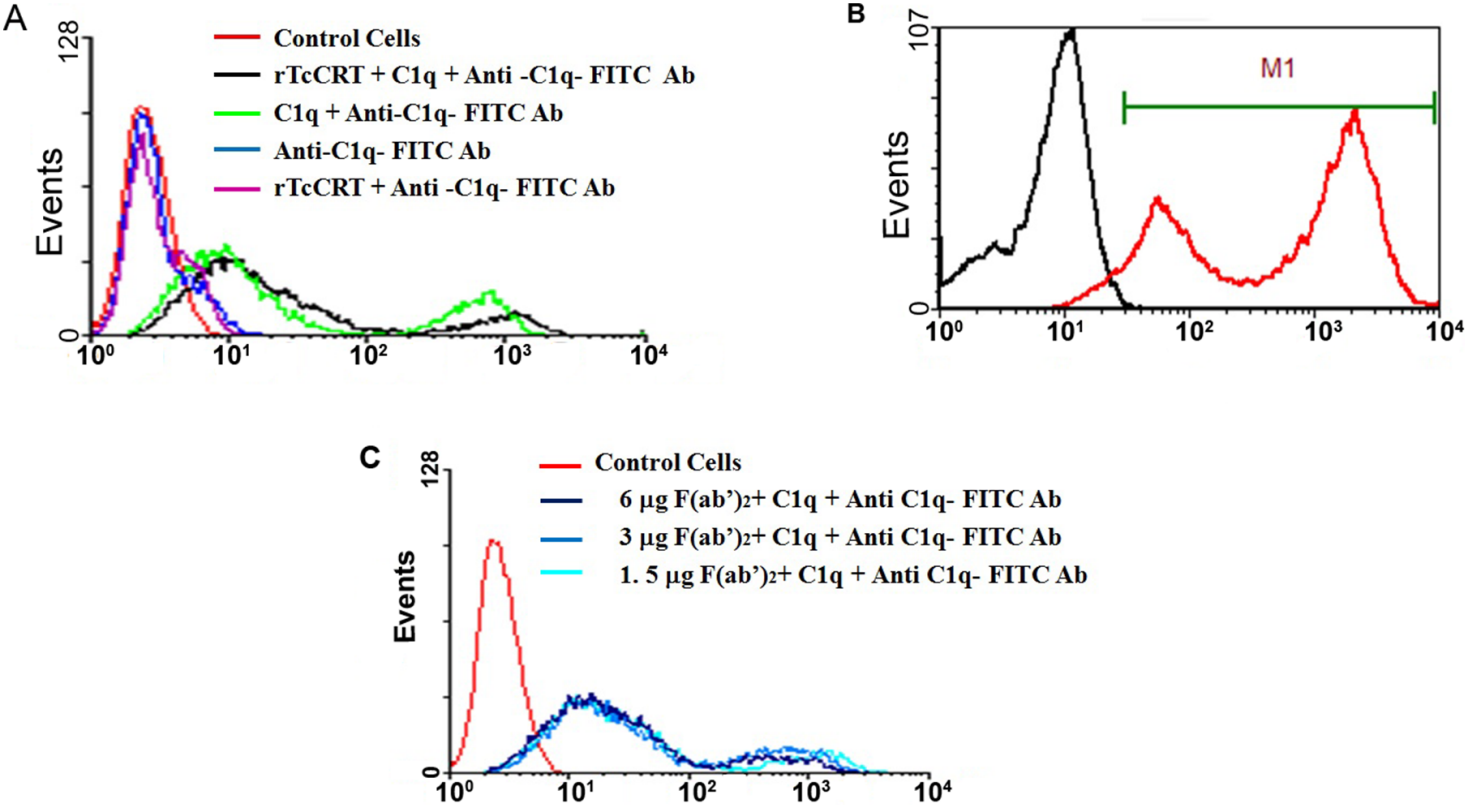
C1q, the first component of the complement system, binds to melanoma cells. By flow cytometry: A: C1q binds to B16-F10 cells in the presence of rTcCRT. B: B16-F10 melanoma cells express murine Calreticulin on their membrane. C: Different concentrations of F(ab’)_2_ anti-rTcCRT antibody fragments do not inhibit the C1q binding.

Since CRT upon translocation to the cell surface in *T. cruzi* or mammal cells (tumors included) captures C1, it was relevant to test whether C1q adheres to the membrane of B16-F10 melanocytes. This was indeed the case, even in the absence of rTcCRT (Figure 5A). MmCRT on the cell surface likely explains such binding (Figure 5B), since F(ab’)_2_ anti-rTcCRT antibody fragments [38] were unable to inhibit this interaction, as shown in Figure 5C.

### C1q and rTcCRT, Applied Individually or Together, Enhanced Phagocytosis of B16-F10 Melanoma Cells by RAW 264.7 Murine Macrophages

**Figure 6:**
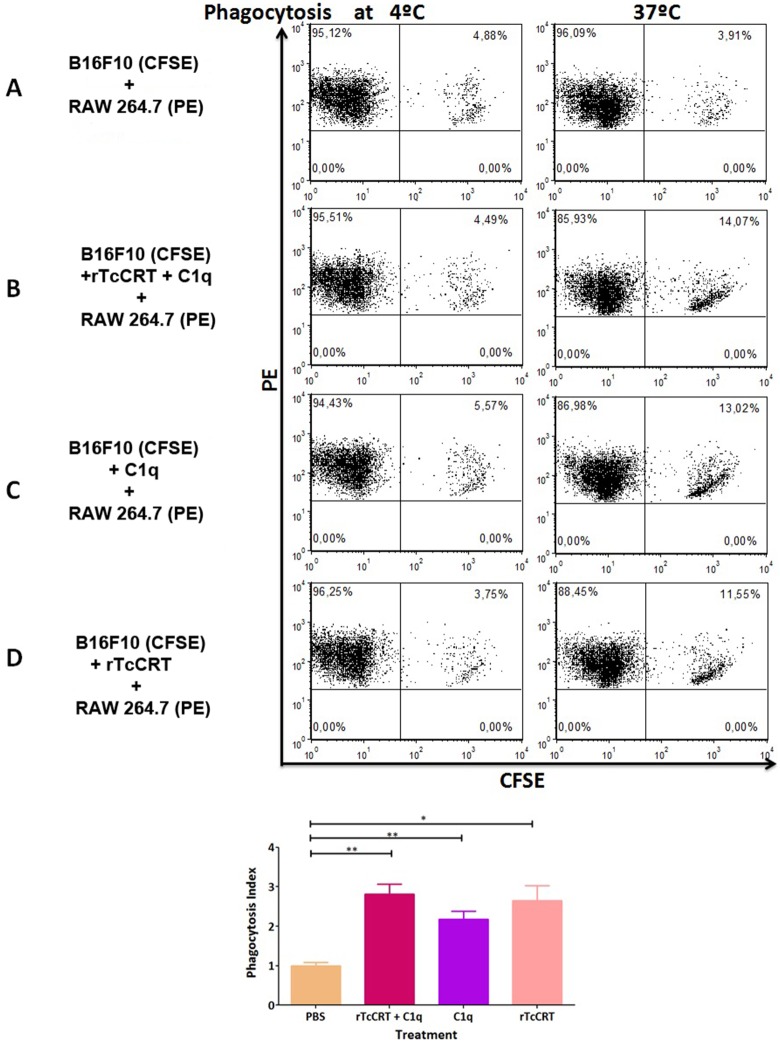
The presence of rTcCRT or C1q enhances phagocytosis of B16-F10 cells by RAW 264.7 macrophages. A: In the simultaneous absence of rTcCRT and C1q, basal, non-temperature dependent phagocytosis is observed. B: Phagocytosis when both, rTcCRT and C1q molecules are simultaneously present. Tumor cells are phagocytized, in a temperature-dependent fashion, when either recombinant C1q (C) or TcCRT (D) are exogenously added. E: Phagocytosis quantification. Statistical analysis by t test (*p = 0.0146; **: p = 0.0029 to 0.0064).

Tumor cells were phagocytized, in a temperature-dependent fashion, when either C1q (Figure 6C) or recombinant TcCRT (Figure 6D) were exogenously added. In the absence of rTcCRT or C1q, only basal, non-temperature dependent phagocytosis was observed (Figure 6A). Conversely, a similar degree of phagocytosis was observed when both exogenous molecules were simultaneously added (Figure 6B). Phagocytosis was quantified and statistically validated in Figure 6E.

### rTcCRT Inoculation Promotes a Humoral Immune Response that is Stimulated by *pSurv,* in Melanoma-bearing Mice

**Figure 7:**
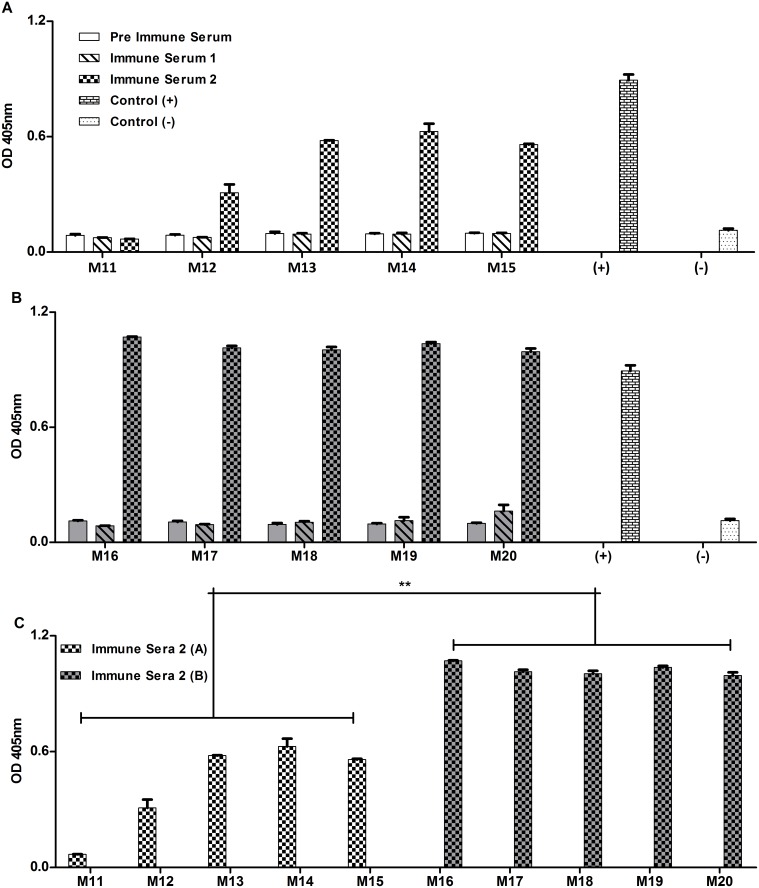
rTcCRT subcutaneously inoculated generates specific antibodies and *pSurv* stimulates its immunogenicity. Pre-immune sera (obtained prior to *pSurv* immunization and rTcCRT administration) and immune sera, obtained at 7 and 21 days after the first rTcCRT inoculation (Immune Sera 1 and 2, respectively), were evaluated by ELISA. Control (+): Wells coated with rTcCRT detected by a specific monoclonal antibody against the parasite protein. Control (–): Wells coated with BSA detected by a specific monoclonal antibody against rTcCRT. A: rTcCRT, inoculated s.c., without adjuvants, induces antibodies. B: Genetic immunization with *pSurv,* followed by rTcCRT administration, improves the production of IgG anti-rTcCRT. C: A *versus* B. Statistical validation of the *pSurv* humoral immune stimulating effect (**: p = 0.004).

TcCRT is known to be immunogenic both in humans and mice [39]–[41]. The *in vivo, ex vivo* or *in vitro* presence of anti-rTcCRT antibodies has also been known to increase C1q recruitment [18], [29], [42], [43]. A possible consequence could be complement activation directed against tumor cells, among other potential cell destructive activities. Indeed, rTcCRT inoculated s.c. without adjuvants, was immunogenic in mice (Figure 7A). Genetic immunization with *pSurv,* followed by rTcCRT administration, induced the production of higher levels of IgG anti-rTcCRT (Figure 7B *versus* 7A). In Figure 7C the humoral immune stimulating effect of *pSurv* is statistically validated.

## Discussion

In this study, we show that immunization with human Surv, together with systemic rTcCRT administration, induces synergistic inhibitory effects on tumor growth in a murine melanoma model. This result is attributed to the combination of a humoral immune response together with enhanced phagocytosis.

The rationale supporting the experimental tumor treatment with *Surv* together with rTcCRT was based on the following data, published by our and other laboratories [3], [11], [21], [24], [31], [44]–[48]: **i).** Surv is over expressed in tumors, **ii).** *Surv* genetic immunization generates an adaptive specific cellular immune response, against tumor cells and/or endotheliocytes, **iii).** TcCRT displays anti-angiogenic and anti-tumor activity *in vivo*, and it is more efficient than HuCRT at equimolar terms, **iv).** Stressed tumor cells translocate mammalian CRT to their membranes, **v).** Cell surface CRT captures Complement C1 (phagocytosis signal) and, **vi).** C1q, present on the membrane of tumor cells, is recognized by dendritic cells leading to phagocytosis followed by antigen processing and generation of an anti-tumor immune response. Based on these data, we evaluated the efficacy of a plasmid encoding Surv (*pSurv*) in conjunction with rTcCRT to inhibit tumor growth.

Synergism is evident when *pSurv* genetic immunization was utilized in conjunction with rTcCRT, which results in significant growth inhibition of an experimental murine melanoma (Figure 1). Interestingly, the *pSurv*-rTcCRT treated group reduced its tumor growth rate, as compared with the group treated with rHuCRT, alone or combined with *pSurv*. This indicated that the parasite-derived molecule is more efficient than the product of its human orthologous gene in the induction of the anti-tumor activity, a fact already described by us [31]. In agreement with these observations, the time necessary for tumors to reach the 3,000 mm^3^ (maximum volume allowed by current bioethical regulations) is longer in *pSurv*-rTcCRT treated animals (Figure 1C).

The rTcCRT anti-angiogenic/anti-tumor effect has been restricted to the 1–193 N-terminal domain. Since between the N and R domain (aa 136–281) there is an overlap of only 57 aa [49], we asked whether the R domain might serve as a negative control for the N-domain mediated anti-angiogenic effect. However, the R domain retains the anti-tumor activity (Figure 2). Similar to the whole protein, the R-domain combined with *pSurv* immunization delays tumor growth (Figure 2C). More precise mapping of the anti-angiogenic/anti-tumor activity residing in this 57 aa sequence, present in both the R and N domains, is currently underway in our laboratory.

Angiogenesis is required for nutrition, oxygen and waste disposal in tumors larger than 2 to 3 mm [50]. Analysis of tumor tissues may distinguish various histological features of this melanoma, like dermis invasive capacity and progress towards the large amount of connective tissue in the parenchyma. Many, very tortuous, small caliber blood vessels are found (data not shown). By immunohistochemistry with an anti-CD31 antibody we determined that rTcCRT administration or *pSurv* immunization, alone or combined, were anti-angiogenic (Figure 3). Despite the common outcomes with these experimental treatments, the molecular targets and mechanisms involved are different. Endothelial cells transiently over express Surv in their proliferative period [7], [8]. If Surv is immunogenic, its immune recognition on endothelial cells may result in anti-angiogenicity and thus, anti-tumor effects. Although, on its own, rTcCRT does not affect tumor growth, it inhibits endothelial cell proliferation, capillary morphogenesis and development [31]. We therefore propose that the anti-tumor effect of this parasite molecule is mainly due to its anti-angiogenic properties.

Previous publications describe the murine CRT translocation to the B16 cell membrane [51]. However, it was also possible for us to bind rTcCRT to the tumor cells, in concentrations different from those used in the *in vivo* assays (Figure 4). Perhaps, translocation of the molecule may be enhanced in response to stressful stimuli and this is the reason for the basal binding of fluid phase C1q to B16-F10 cells, even in absence of exogenously added rTcCRT (Figure 5). As expected, this interaction was not reversed by F(ab’)_2_ antibody fragments specific against the parasite molecule (Figure 5).

At present it is known that CRT is homologous to translocated mammalian cC1qR, a complement C1 receptor present on a variety of biological membranes (*i.e*. macrophages) [24]. Thus, when tumor cells were pre-incubated with rTcCRT and then with C1q, they interacted with macrophages most likely via cC1qR (Figure 6B), with subsequent phagocytosis. However, this was also observed when phagocytic cells were incubated only with C1q (Figure 6C) or rTcCRT (Figure 6D). In these experiments, alternative sources of CRT [21] or C1q [52] may be the tumor cells or macrophages, respectively, both present in these *in vitro* assays. No phagocytosis, beyond the basal level, was obtained when these cells where co-cultured in the *absence of both* exogenous CRT and C1q. Perhaps, in this situation phagocytosis was only mediated by endogenous C1q and MmCRT molecules, at essentially basal levels (Figure 6A). Other studies described also the CRT as an emerging immune-relevant molecule associated with host immune responses, *i.e*., the amphioxus *Branchiostoma japonicum* binds to *Escherichia coli* and to *Staphylococcus aureus* and promoted phagocytosis by macrophages [53].

By unknown mechanisms, rTcCRT was immunogenic, in the absence of adjuvants, in treated mice, especially in those previously immunized with *pSurv* (Figure 7). Among other possibilities, these antibodies may interfere with the anti-angiogenic TcCRT effect, by intervening in the interaction of the parasite molecule with its receptors on endothelial cells. The second bleeding, in which we detected anti-rTcCRT antibodies, was obtained on day 21, after eight rTcCRT inoculations, the last day of tumor formation assay. However, specific anti-rTcCRT antibodies may already be available at day 7 (first bleeding) and increase subsequently to the level found on day 21. Recognition of melanoma bound rTcCRT, by anti-rTcCRT antibodies may promote additional Fc-dependent phagocytosis, complement activation, further accumulation of C1q (‘eat me signals’), among other possibilities. On the other hand, the immune complexes formed may be taken up by antigen presenting cells (B cells, macrophages and dendritic cells), with resulting enhanced anti-rTcCRT humoral immune response.

Responses of different tumor types to the large number of available experimental therapies are extremely variable. Therefore, it remains to be determined whether the proposed experimental anti-tumor protocols described herein will be effective in other tumor models. In our perception, it is still premature to propose a translational value for the findings described herein. We will soon start animal experimental combinations of surgical therapies combined with the protocols now proposed. Contingent to these results, clinical assays in humans should first address the stringent bioethical rules of our country.

**Figure 8:**
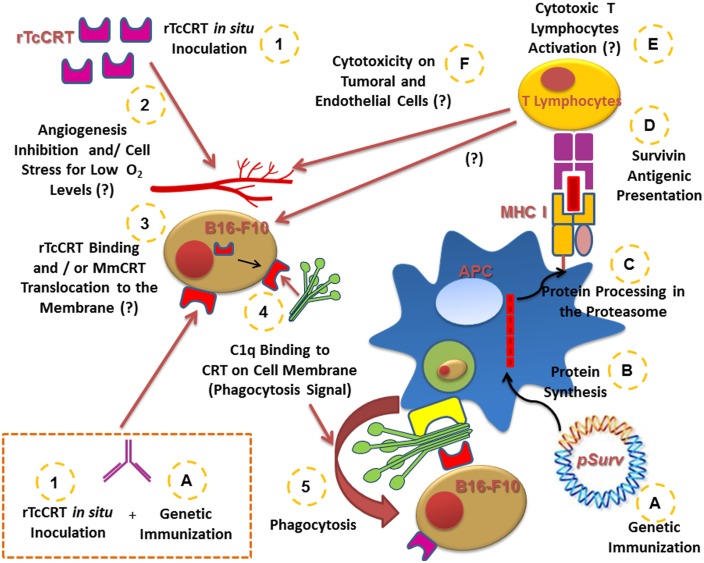
Proposed synergic effect of genetic immunization with Survivin, in conjunction with administration of recombinant TcCRT. rTcCRT (1), inhibits angiogenesis and thus tumor growth. The consequent lack of nutrients and oxygen generate stress on tumor cells (2). This could mediate translocation of CRT murine tumor cell (MmCRT) to the surface (3). rTcCRT and/or MmCRT recruits C1q, (4), acting as a signal for phagocytic antigen presenting cells (APC) (5). Moreover, *pSurv* immunization (A) generates the protein synthesis by the APC (B), processing it in the proteasome (C) and presenting on MHC I molecules (D) and consequent, cytotoxic T lymphocyte activation (E) which may act on the endothelial cell (by inhibiting angiogenesis) and/or on the tumor cells (F). Finally, the combination of both treatments generates the humoral response against rTcCRT in mice (see text for possible consequences). Question marks indicate some purely hypothetical possibilities.

Figure 8 summarizes the results presented here and also includes some hypothetical considerations. Combining rTcCRT parenteral administration with *Surv* genetic immunization synergistically interferes with the growth of a B16-F10 murine melanoma, by eliciting a variety of mechanisms, such as increasing tumor cell phagocytosis and enhancing anti-tumor cell immune responses, among other possibilities.
